# Two Cases of Posttraumatic *Kosakonia* Infection, Argentina, 2023

**DOI:** 10.3201/eid3203.251714

**Published:** 2026-03

**Authors:** Claudia Barberis, Maria Sol Haim, Paula Zomero, Germán Traglia, Alejandro Ellis, Roxana Cittadini, Tomás Poklépovich, Marisa Almuzara, Carlos Vay

**Affiliations:** University of Buenos Aires, Faculty of Pharmacy and Biochemistry, “Hospital de Clínicas José de San Martín”, Buenos Aires, Argentina (C. Barberis, P. Zomero, M. Almuzara, C. Vay); National Center for Genomics and Bioinformatics Unit, ANLIS “Dr. Carlos G. Malbrán”, Buenos Aires (M.S. Haim, T. Poklépovich); CENUR Litoral Norte Genomics and Bioinformatics Unit, University of the Republic, Salto, Uruguay (G. Traglia); Sanatorio Mater Dei, Buenos Aires (A. Ellis, R. Cittadini, C. Vay)

**Keywords:** bacteria, *Kosakonia* spp., human infection, emerging pathogen, Argentina

## Abstract

We describe 2 plant-associated posttraumatic *Kosakonia* infections in Argentina. Facing biochemical and matrix-assisted laser desorption/ionization time-of-flight mass spectrometry limitations, we used whole-genome sequencing to successfully identify *K. cowanii* and *K. oryzae* as the causative agents. Our data highlight the crucial role of genomics in correctly identifying these underestimated emerging pathogens.

Medical literature recognized the genus *Kosakonia* in 2013, after the systematic reorganization of *Enterobacter* genus (*1*). Largely known as plant growth–promoting bacteria, or phytopathogens (*2*), the species included in this genus are rapidly gaining relevance as opportunistic human pathogens. However, because of the bacteria’s phenotypic similarities with *Enterobacter* and *Pantoea*, clinicians frequently misidentify *Kosakonia* infections, leading to an underestimation of their true clinical incidence (*3*–*5*). We describe 2 cases of osteomyelitis in Argentina caused by *Kosakonia* species associated with environmental trauma.

Case 1 involved a 12-year-old girl with an open supracondylar elbow fracture sustained falling from a horse and involving soil contamination. Despite surgical fixation and cephalosporin prophylaxis, she sought treatment 10 days later for purulent discharge. Cultures yielded a gram-negative rod (isolate CMVA41). We treated the suspected osteomyelitis with intravenous piperacillin/tazobactam and clindamycin, followed by oral ciprofloxacin and clindamycin for 6 weeks, resulting in complete resolution.

Case 2 involved a 20-year-old man with chronic posttraumatic knee osteomyelitis following a puncture with a tree thorn. We cultured a gram-negative rod (isolate CMVA47) from surgical samples. We administered vancomycin and piperacillin/tazobactam, followed by a course of oral amoxicillin and ciprofloxacin for 6 weeks, achieving clinical cure.

We performed a polyphasic identification approach for both cases. Colonies were yellow and lactose-fermenting on eosin methylene blue (Levine) agar. Initial phenotypic identification using conventional biochemical tests failed to provide reliable genus-level identification (Appendix Table). We subsequently performed matrix-assisted laser desorption/ionization time-of-flight (MALDI-TOF) mass spectrometry (Bruker Biotyper, library v13.0; https://www.bruker.com). We identified isolate CMVA47 as *Kosakonia cowanii* with a secure species-level score (2.052). In contrast, we initially misidentified isolate CMVA41 as *K. radicincitans* with a low confidence score (1.717), indicating probable genus-level identification but species uncertainty. This result highlighted a limitation: the spectral library lacked a reference profile for *K. oryzae*, leading to potential misclassification (*6*).

To resolve those uncertainties, we performed whole-genome sequencing (WGS) using the Illumina NovaSeq6000 platform (https://www.illumina.com). For strain CMVA41, the 16S rRNA gene sequence showed 100% identity with the reference *K. oryzae* sequence Ola 51. Relative to the same reference, we noted an average nucleotide identity value of 98.83% and a digital DNA-DNA hybridization value of 91.6%, with ribosomal multilocus sequence typing identifying the strain as the same species with 94% support. Those results differed from MALDI-TOF mass spectrometry identification and indicated *K. oryzae* as a clinically relevant human pathogen. For strain CMVA47, although the 16S rRNA gene (99.77% identity) and average nucleotide identity (95.94%) supported identification as *K. cowanii*, the digital DNA-DNA hybridization value (65.1%) fell below the 70% threshold typically used for species delineation.

We produced a core genome phylogeny analysis based on concatenated sequences of 3,232 core genes that shared >50% sequence identity and were present in >80% of the included genomes (1 reference genome of each *Kosakonia* species, if present, downloaded from the National Center for Biotechnology Information RefSeq database [https://www.ncbi.nlm.nih.gov/refseq]). CVMA41 clustered with *K. oryzae* and CVMA47 clustered with *K. cowanii*, with 100% bootstrap support (Figure). The genomic divergence observed in CMVA47 suggested that revisiting genomic thresholds within the *Kosakonia* genus might be necessary, as seen in other genera (*7*).

**Figure:**
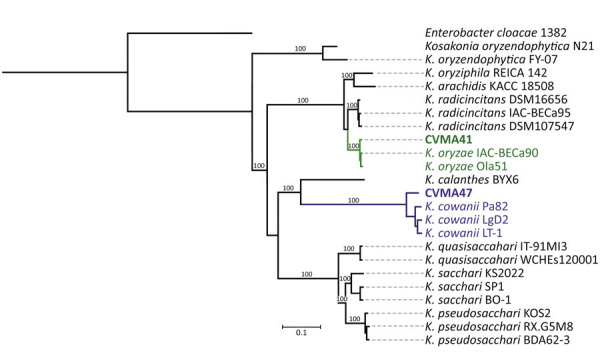
Data from a study of 2 cases of posttraumatic *Kosakonia* infection, Argentina, 2023. Maximum-likelihood phylogeny calculated using 1,214,977 single-nucleotide variants from a core-gene alignment of 3,232 genes from genomes of *Kosakonia*-described species with 1,000 bootstraps. Green indicates CVMA41 clusters, violet indicates CVMA47 clusters. Tree rooted in an *Enterobacter cloacae* genome included as an outgroup. Scale bar indicates substitutions per site.

Antimicrobial susceptibility testing revealed that both isolates were susceptible to aminoglycosides, fluoroquinolones, trimethoprim/sulfamethoxazole, extended-spectrum cephalosporins, and carbapenems. Of note, although CMVA41 was susceptible to all tested agents, CVMA47 exhibited resistance to ampicillin and intermediate susceptibility to cefazolin. This phenotypic profile serves as a marker distinguishing *Kosakonia* spp. from *Enterobacter cloacae* complex. *Enterobacter cloacae* complex typically exhibits intrinsic resistance to ampicillin/sulbactam and carries an inducible chromosomal *ampC* β-lactamase that can lead to third-generation cephalosporin resistance upon derepression, but both *Kosakonia* isolates remained susceptible to these agents. Genomic analysis confirmed the absence of *ampC* and its regulator *ampR* in both strains. This distinction is clinically relevant, supporting the use of ampicillin/sulbactam or cephalosporins as therapeutic options, sparing carbapenems. Consequently, we theorized that the ampicillin resistance in CVMA47 was likely attributable to the putative chromosomic β-lactamase KSA-1, which was identified in *Kosakonia sacchari*, showing 78.6% similarity to this class A extended-spectrum β-lactamase, rather than an AmpC-type enzyme (*8*). Our results further support that genomic divergence in CVMA47 is biologically meaningful. Researchers noted similar findings regarding *Kluyvera* spp., where the presence of intrinsic β-lactamases were linked to species differentiation and the proposal of refined taxonomic thresholds (*9*).

Identifying *Kosakonia* isolates in this study illustrates the challenges that clinical laboratories face with emerging pathogens. The cases we describe contribute to the growing evidence that *Kosakonia* infections are strongly associated with traumatic inoculation of plant material, although reports have described endogenous infections (*3*,*10*). The identification of *K. oryzae* as a human pathogen expands the spectrum of *Kosakonia* species with clinical relevance. MALDI-TOF mass spectrometry represents a considerable improvement over biochemical tests, but the reliability of such analysis is contingent on updated databases (*6*). WGS thus stands as the standard for accurate identification of such environmental pathogens, essential for defining their epidemiology and guiding antimicrobial stewardship.

Appendix Additional information for two cases of posttraumatic *Kosakonia* infection, Argentina, 2023.
