# Supplementary material for: Two Cases of Posttraumatic Kosakonia Infection, Argentina, 2023
Source: Emerg Infect Dis. 2026 Mar;32(3):459–62. doi: 10.3201/eid3203.251714 (PMC13016020; doi:10.3201/eid3203.251714)
Supplement: Appendix — Additional information for two cases of posttraumatic Kosakonia infection, Argentina, 2023. [file 25-1714-Techapp-s1.pdf]

Article DOI: http://doi.org/10.3201/eid3203.251714

EID cannot ensure accessibility for supplementary materials supplied by authors. Readers who have difficulty accessing supplementary content should contact the authors for assistance.

Two Cases of Posttraumatic *Kosakonia* Infection, Argentina, 2023

Appendix

Appendix Table. Biochemical characteristics of *Kosakonia* spp. isolates and reference strains from a study of 2 cases of posttraumatic *Kosakonia* infection, Argentina, 2023*

Test	Isolate CVMA41	Isolate CVMA47	*K. oryzae* (1)	*K. cowanii* (2)	*K. radicincitans* (3)
TSI Agar	A/A gas no H_2S	A/A gas no H_2S	A/A gas no H_2S	A/A gas no H_2S	A/A gas no H_2S
Oxidase	-	-	-	-	-
Motility	+	+	+	+	+
Citrate	+	+	+	+	+
Indole	-	-	-	-	-
ADH	+	-	+	-	+
ODC	-	-	-	-	-
LDC	-	-	-	-	-
VP	+	+	+	+	+
Esculine	-	+	-	+	+
Gelatine	-	-	ND	-	-
ONPG	+	+	ND	+	+
DNase	-	-	ND	-	-
Urease	-	-	ND	-	-
PDA	-	-	ND	-	-
Malonate	+	-	+	-	+
Melibiose	-	+	ND	+	-

*TSI: triple sugar iron, VP: Voges Proskauer, ADH: arginine dehydrolase, ODC: ornithine decarboxylase, LDC: lysine decarboxylase, PDA: phenylalanine deaminase; A/A: Acid/acid, +: positive, -: negative, ND: not available.

References

1. Brady CL, Venter SN, Cleenwerck I, Engelbeen K, de Vos P, Wingfield MJ, et al. Isolation of *Enterobacter cowanii* from Eucalyptus showing symptoms of bacterial blight and dieback in Uruguay. Lett Appl Microbiol. 2009;49:461–5. PubMed https://doi.org/10.1111/j.1472-765X.2009.02692.x

2. Inoue K, Sugiyama K, Kosako Y, Sakazaki R, Yamai S. Enterobacter cowaniisp. nov., a new species of the family Enterobacteriaceae. Curr Microbiol. 2000;41:417–20. PubMed https://doi.org/10.1007/s002840010160

3. Kämpfer P, Ruppel S, Remus R. Enterobacter radicincitans sp. nov., a plant growth promoting species of the family Enterobacteriaceae. Syst Appl Microbiol. 2005;28:213–21. PubMed https://doi.org/10.1016/j.syapm.2004.12.007
